# Persistence of a sessile benthic organism promoted by a morphological strategy combining sheets and trees

**DOI:** 10.1098/rspb.2022.0952

**Published:** 2022-07-13

**Authors:** Peter J. Edmunds

**Affiliations:** Department of Biology, California State University, 18111 Nordhoff Street, Northridge, CA 91330-8303, USA

**Keywords:** coral, reef, Caribbean, *Millepora*, phenotypic plasticity

## Abstract

Sessile organisms exploit a life-history strategy in which adults are immobile and their growth position is determined at settlement. The morphological strategy exploited by these organisms has strong selective value, because it can allow beneficial matching of morphology to environmental and biological conditions. In benthic marine environments, a ‘sheet-tree’ morphology is a classic mechanism exploited by select sessile organisms, and milleporine hydrocorals provide one of the best examples of this strategy. Using 30-year analysis of *Millepora* sp. on the reefs of St. John, US Virgin Islands, I tested for the benefits of a sheet-tree morphology in mediating the ecological success of an important functional group of benthic space holders. The abundance of *Millepora* sp. chaotically changed from 1992 to 2021 in concert with hurricanes, bleaching and macroalgal crowding. *Millepora* sp. responded to these disturbances by exploiting their morphological strategy to increase the use of trees when their sheets were compromised by bleaching and spatial competition with macroalgae, and the use of sheets when their trees were broken by storms. Together, these results reveal the selective value of a plastic sheet-tree morphology, which can be exploited by sessile organisms to respond to decadal-scale variation in environmental conditions.

## 1. Introduction

Sessile organisms are confronted by unique circumstances arising from their growth in a position determined at settlement [1,2]. Following establishment, success is mediated by prevailing conditions, and when these change, the capacity to respond through phenotypic plasticity has strong selective value [3]. Although sessile taxa are ubiquitous in most biomes [1,4], they are particularly well known in marine benthic habitats where morphology has long been recognized as a plastic trait contributing to fitness [1,5]. In these habitats, anthropogenic effects are causing large changes in community structure, as well as the environmental conditions to which they are exposed [6]. Confronted with these challenges, the morphological strategies of sessile community members may become an important means to improve fitness under present and future conditions [1,5].

The morphological strategies of sessile marine organisms are critical features determining evolutionary success, and 43 years ago, a seminal contribution [1] described six strategies that could be exploited individually and in combination. Most of these have remained in the theoretical domain without rigorous empirical support. Several combinations of the six strategies [1] create unusual opportunities, with one combination of ‘sheets’ and ‘trees’ that can be produced in the same individual. In a ‘sheet-tree’ morphology, sheets arise as thin layers of tissue and skeleton over the substratum and occur in at least 10 taxa; trees arise through the production of branches of variable length and flexibilities reflecting high commitment to survival at the site of settlement and exploitation of the overlying seawater, and they occur in at least 11 taxa (on Caribbean reefs [1]). Sheet-tree organisms were hypothesized to flourish under diverse conditions through the use of plastic transitions between morphologies [1, p. 545]. Examples of this strategy have remained rare, and only a bryozoan example [7] originally was provided [1]. Morphological strategies similar to sheet-trees can be found among fungi [8], trees [9], slime moulds [10], flowering plants [11], and potentially in the fronds and basal discs of the rangeomorph biota of the Ediacaran period [12]. Among marine sessile taxa, one of the best examples of a sheet-tree morphology is provided by the calcareous hydrocoral, *Millepora* spp. [13,14].

*Millepora* spp. are hermatypic corals that are globally distributed and represented by 16 species [15]. They share features with scleractinians, including a colonial modular design, rapid calcification, a mutualistic symbiosis with symbiodiniacean algae and polytrophic nutrition with resources acquired through autotrophy and heterotrophy [13,14]. In contrast with scleractinians (Anthozoa), *Millepora* spp. are hydrozoans with pores instead of corallites in which polyps are located, and they produce medusae asexually, which then release sexual gametes soon after emergence. Syngamy generates pelagic larvae, the settlement of which determines where adult colonies are located [14]. *Millepora* spp. produce morphologically complex colonies with sheets spreading across the substratum and upright trees having a diversity of shapes and sizes [13,14,16]. The morphology of *Millepora* spp. is dependent on environmental conditions, particularly the flow regime [13,14,17], but under extreme high flow, branches are detached [18] and function as asexual propagules.

Since milleporine hydrocorals are found in the fossil record extending to the Cretaceous (approx. 84 Ma [19]) and are ubiquitous on present-day reefs [13,14], it is reasonable to infer that they are ecologically successful. This assertion is consistent with examples of high historic abundances (e.g. 26–40% cover [20,21]), their abundance on most reefs, albeit at modest cover and in select zones (e.g. less than 10% in the Western Atlantic [13]), and their capacity to function as a hermatype [13,14]. Yet the reefs on which *Millepore* spp. now grow are ecologically and physically different from those of a few decades ago [22]. In the Caribbean, scleractinian cover is reduced [23], macroalgae are highly abundant [24], seawater is warmer [25], hurricanes are more intense [26], and diseases are common [27]. These changes present opportunities and challenges to *Millepora* spp., potentially favouring the spread of their sheets over space formerly occupied by scleractinians. Opposing this trend, the growth of *Millepora* spp. sheets probably is impeded through competition with macroalgae [28] and bleaching [14], thereby favouring the growth of trees to escape from the benthos [1], at least until they are broken by storms [18]. As surmised by Jackson [1], the sheet-tree morphology of *Millepora* spp. may be well suited for ecological success in what are becoming novel ecosystems [29].

This study describes the population dynamics and morphology of *Millepora* spp. over three decades, and the results are used to test for the functional significance of a sheet-tree morphology [1] in a crowded and disturbed habitat. First, I tested the hypothesis that the abundance and morphological strategy (i.e. quotients of tree abundance (number of branches) to sheet area) of *Millepora* spp. has changed. Second, I interpreted the outcome of testing this hypothesis within the framework of generalized additive models (GAMs) to identify the morphological response to spatial competition (with macroalgae), thermal stress (leading to bleaching) and physical disturbances (hurricanes). These analyses were rationalized by the effects of spatial competition and bleaching in reducing the areas of sheets, thus favouring trees (i.e. branches), and the effects of hurricanes in removing branches, thereby increasing the ecological value of sheets.

## 2. Material and methods

This study was completed at 9 m depth using photoquadrats (0.5 × 0.5 m) recorded annually along a transect (20–40 m long) at Cabritte Horn (18.3075° N, −64.7219° W) on the south shore of St John, US Virgin Islands. This rocky headland is locally known for rough seawater conditions, and flow speeds of approximately 1 m s^−1^ (12 m depth) to approximately 2.5 m s^−1^ (4 m depth) have been recorded near the present site over 48 h of ‘rough seas’ in April [30], and undoubtedly are much greater during hurricanes. The transect was permanently marked and first sampled on 30 May 1992 with a mean sampling date of 19 July (with a ±95% CI of 7 days) (electronic supplementary material, table S1). Photoquadrats were recorded using cameras mounted on a framer approximately 80 cm above the reef. Colour slide film was used from 1992 to 2000 (and digitized at 4000 dpi), with digital photography from 2001 (electronic supplementary material, table S1).

At each sampling, photoquadrats were recorded at random positions along the transect, which was 20 m long from 1992 to 1999 (*n* approx. 17 photoquadrats y^−1^), and 40 m from 2000 (*n* approx. 40 photoquadrats y^−1^). Photoquadrats were used to determine the percentage cover of benthic space holders using CoralNet software [31] with 200 random points on each image that were manually annotated. All aspects of the benthic community were quantified [32], but here only the cover of macroalgae is statistically compared among years because of its role in spatial competition with *Millepora*. Photoquadrats also were used to quantify *Millepora* abundance using ImageJ software [33], and *Millepora* (here, *M. alcicornis, M. complanata* and *M. squarosa*) were resolved to the species complex consisting of *M. alcicornis* and *M. complanata* [34]. As 99.8% of colonies were *M. alcicornis* (described in results, and representative of this region), colonies were considered a single species and described as ‘*Millepora* sp’. Areas of encrusting *Millepora* sp. were located in each photoquadrat, outlined, and their areas measured with all pieces of sheets quantified separately. Because *Millepora* sp. sheets meander across the substratum and are prone to fission, it was not always clear where colonies began and ended within photoquadrats. Colonies therefore were defined as areas of autonomous tissue, and portions of colonies that were partially within the photoquadrats were scored as separate colonies (such cases were relatively uncommon).

**Figure 1.**
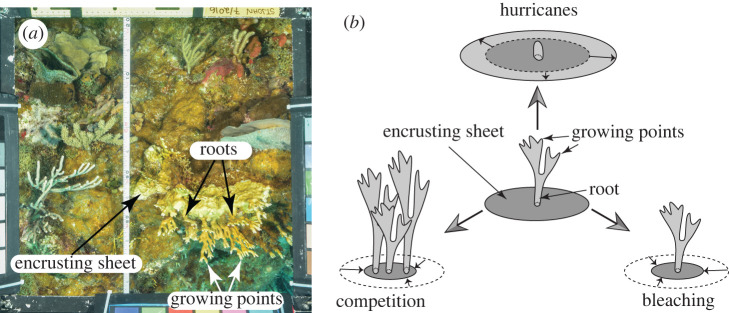
Representative quadrat (0.5 × 0.5 m) showing *M. alcicornis* (*a*), and a schematic of the relationships between trees and sheets of *Millepora* sp. and how they change in response to environmental conditions (*b*). In (*a*), roots are proximal origins of branches on sheets, and growing points are the distal apices of branches. In (*b*), hurricanes remove branches but leave sheets intact, competition encroaches upon sheets and bleaching reduces sheet area through mortality. (Online version in colour.)

Branches on each sheet were quantified as the number of ‘roots’ where they attached to the sheet, and as the number of growing points on each root (figure 1). Branch fragments on the benthos were not counted. The size of *Millepora* sp. colonies was determined from the mean planar area of autonomous portions of sheets (cm^2^), and roots and growing points were normalized to the sheet (roots 100 cm^2^ and growing points 100 cm^2^) and colony (roots colony^−1^ and growing points colony^−1^); growing points were also expressed per root (growing points root^−1^). The areas of sheets were summed by quadrat to calculate the percentage cover of *Millepora* sp. Occasionally *Millepora* sp. was found encrusting octocorals [35] on which they appeared as long branches with miniscule sites of basal attachment; such colonies were excluded from analyses of roots and growing points.

Seawater temperature was measured with loggers (mostly Onset Computer Corp., Hobo U22-001, ±0.2°C) sampling at 0.0011 Hz and located approx. 900 m from Cabritte Horn at Yawzi Point [36]. Temperature was averaged by day and month to characterize the mean of the hottest three months prior to each sampling (temperature^1^, August–October, mean ± s.e., *n* = 3), and between 31 July and the previous 1 August by study year (temperature^2^, mean ± s.e., *n* = 12 months). Rainfall from 1992 to 2011 was obtained from the Southeastern Regional Climate Center (https://sercc.com/), which compiled data from a rain gauge in Cruz Bay, St John (Station 671980). Where this record was incomplete, values were obtained from Catherinburg (Station 671348), East End (Station 672551) or through interpolation [36]. From 2012, rainfall was measured using a Standard Rain Gauge (NOAA, National Weather Service) deployed on the north shore (18.3558° N, −64.7660° W) (station VI-SJ-3, https://wys.cocorahs.org). Rainfall was summarized by calendar year (cm y^−1^) and used in the present analyses summarized from 31 July to the previous 1 August by study year.

### (a) Statistical analyses

Macroalgal cover (arcsine transformed), the morphology of *Millepora* sp. (log-transformed) and temperature were compared among years using one-way ANOVAs with Bonferonni *post hoc* analyses to compare between years (using Systat 13 software). The capacity of *Millepora* sp. to exploit a sheet-tree morphology was quantified through the quotient of roots and growing points to the area of the sheet (roots 100 cm^−2^ and growing points 100 cm^−2^). Quotients quantified the exploitation of ‘trees’ relative to ‘sheets’, but they have the limitation of not being able to distinguish between effects caused by the growth of new roots or growing points versus changes in absolute area of the sheets. The relationships between these quotients and environmental conditions were evaluated using GAMs that supported tests for complex nonlinear relationships with multiple predictors. GAMs were prepared using the mgcv package (v. 1.8-34) [37] in R (v. 4.0.5), accessed through the XLSTAT (v. 2021.2.1, Addinsoft, Paris) add-in to Excel 16.54 (Microsoft). Models were run using Gaussian errors, cubic splines and variance components estimated by REML. Models were restricted to three quantitative effects to enhance interpretation [38], and the best model was identified from the lowest corrected Akaike information criterion (AIC_c_) [39].

GAMs employed predictors that captured the effects of hurricanes, bleaching and spatial competition, which affect *Millepora* sp. abundance and morphology [13,14]. The dependent variables (roots 100 cm^−2^ and growing points 100 cm^−2^) were log-transformed to restore normality. Hurricanes were evaluated as a qualitative effect in which years of major hurricanes (Marilyn (1995), Georges (1998), Lenny (1999), Earl (2010) and Irma/Maria (2017)) were assigned a rank of 1, and all other years a zero. Ranks were assigned based on local knowledge and hurricane tracks (https://www.nhc.noaa.gov) to the years preceding the sampling in which effects would first be detected. Bleaching was indirectly evaluated through mean seawater temperature (°C) in the historically hottest months of the year (August–October) prior to sampling (temperature^1^) and the mean temperature in the study year (temperature^2^). Rainfall was summed by study year (cm), and temperature and rainfall were entered as qualitative effects in the GAMs.

## 3. Results

Over 30 years, 1016 photoquadrats were recorded and 51% contained 1153 colonies of *Millepora* sp. Most were *M. alcicornis* (99.8%, including 1% as ‘*M. complanata*’) and 0.2% was identified as *M. squarosa*. Together, these colonies are described as *Millepora* sp.

**Figure 2.**
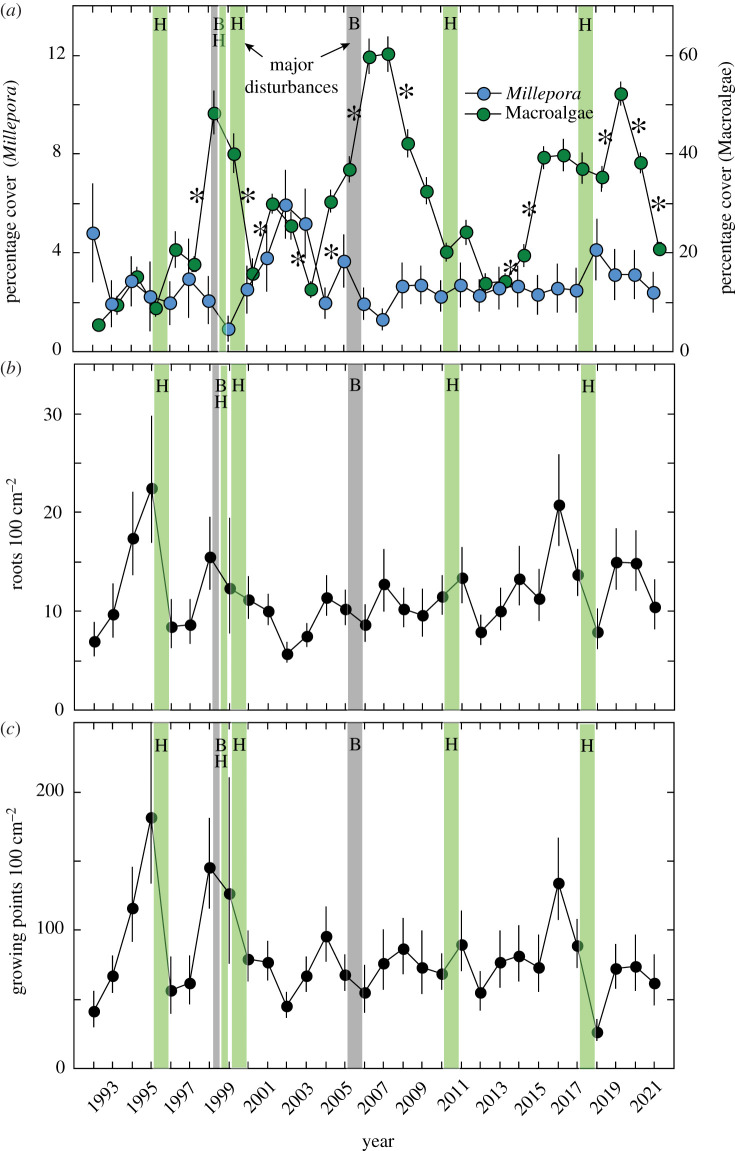
Community structure at Cabritte Horn and features of *Millepora* sp. from 1992 to 2021. (*a*) Mean percentage cover (±s.e., *n* = 16–44) of *Millepora* sp. (left ordinate) and macroalgae (right ordinate), offset on abscissa for clarity. (*b*) Mean (±s.e., *n* = 8–56) abundance of roots per area of sheet. (*c*) Mean (±s.e., *n* = 8–56) abundance of growing points per area of sheet. Mean ± s.e. for roots 100 cm^−2^ and growing points 100 cm^−2^ were determined from log-transformed values that were back-transformed for display. Vertical bars show hurricanes (H, green) and bleaching (B, grey). Asterisk in (*a*) shows significant differences (*p* < 0.05) between consecutive years. (Online version in colour.)

Spatial occupancy by benthic organisms was dynamic, with macroalgae cover varying among years (figure 2*a*). Macroalgae mostly included *Dictyota* spp., *Lobophora* spp. and Peyssonneliaceae (since about 2012), and they varied in cover from 5.5 ± 0.7% in 1992 to 60.3 ± 3.5% in 2007 (mean ± s.e., *n* = 17, 40, respectively), sometimes with large changes between years. Macroalgal cover increased by 30.7% between 1997 and 1998, and declined by 24.3% from 1999 to 2000; large increases accompanied bleaching in 1998 and 2005, but the responses to hurricanes were mixed. Averaged across years, mean macroalgal cover was 28.9 ± 2.7% (±s.e., *n* = 30 year) and changed over time (*F* = 36.745, d.f. = 29,986, *p* < 0.001), with numerous differences among years (figure 2). The cover of crustose coralline algae, algal turf and bare space (combined as CTB) varied from 10.5 ± 2.4% in 1994 to 58.3 ± 4.4% in 2000, and the cover of scleractinians varied from 3.5 ± 0.7% in 2021 to 10.0 ± 2.0% in 2004 (mean ± s.e., results not shown; see [32]).

Sheets of *Millepora* sp. ranged in area from 1 cm^2^ to 927 cm^2^, with a mean (from log-transformed values) of 16 ± 1 cm^2^ (s.e.) (electronic supplementary material, figure S1A). Twenty-five per cent of colonies extended beyond the photoquadrat, and 4% were encrusting dead octocorals; 10% of colonies lacked roots or growing points, and the remainder had 1–77 roots (mean ± s.e. = 3.1 ± 0.1) and 1–159 growing points (21.3 ± 0.6). Based on the summed areas of sheets in the photoquadrats, the mean (± s.e.) cover of *Millepora* sp. ranged from 0.9 ± 0.5% (*n* = 17) in 1999, to 6.0 ± 1.4% (*n* = 44) in 2002, with a grand mean of 2.8 ± 0.2% (±s.e., *n* = 30 year) (figure 2*a*). Large relative declines in mean *Millepora* sp. cover followed bleaching in 1998 (55% reduction) and 2005 (47% reduction), and large relative increases followed hurricanes (168% increase after Lenny in 1999, and 65% increase after Irma and Maria in 2017), and sometimes between years without hurricanes or bleaching (e.g. 87% increase between 2004 and 2005) (figure 2*a*). Overall, the cover of *Millepora* sp. statistically could not be resolved among years (*F* = 0.924, d.f. = 29,986, *p* = 0.583).

The number of roots and growing points per area of sheet, changed among years (figure 2*b,c*). The mean density of roots varied from 5.8 roots 100 cm^−2^ in 2002, to 22.5 roots 100 cm^−2^ in 1995, with a grand mean of 11.6 ± 0.7 roots 100 cm^−2^. The changes over time were significant (*F* = 2.275, d.f. = 29,1056, *p* < 0.001), but *post hoc* pairwise analyses could not distinguish between years (*p* > 0.05). While large declines in the number of roots and growing points followed hurricanes in 1995 and 2017, other large changes in abundance occurred in years without associated hurricanes or bleaching. The mean density of growing points changed in a similar way to roots, varying from 26.9 growing points 100 cm^−2^ in 2018, to 181.5 growing points 100 cm^−2^ in 1995, with a grand mean of 80.7 ± 5.9 growing points 100 cm^−2^ (figure 2*c*). The changes were significant (*F* = 1.740, d.f. = 29,1056, *p* = 0.009), but *post hoc* pairwise analyses could not distinguish between years (*p* > 0.05). Underlying these trends were differences in the number of growing points root^−1^, as well as the number of roots and growing points per colony (electronic supplementary material, figure S1B–D). These changes show that the structure of roots as well as the relationship between sheets and branches changed.

Mean monthly seawater temperature varied from 27.3 ± 0.3°C in 1993, to 28.4 ± 0.3°C in 2020, and increased over time (*F* = 12.892, d.f. = 1,27, *p* = 0.001) at 0.02°C y^−1^ (electronic supplementary material, figure S2). Annualized rainfall varied from 68 cm in 1994, to 194 cm in 2017, but it did not linearly change from 1992 to 2000 (*F* = 2.818, d.f. = 1,27, *p* = 0.105).

### (a) Relationships between sheet-tree morphology and environmental conditions

**Figure 3.**
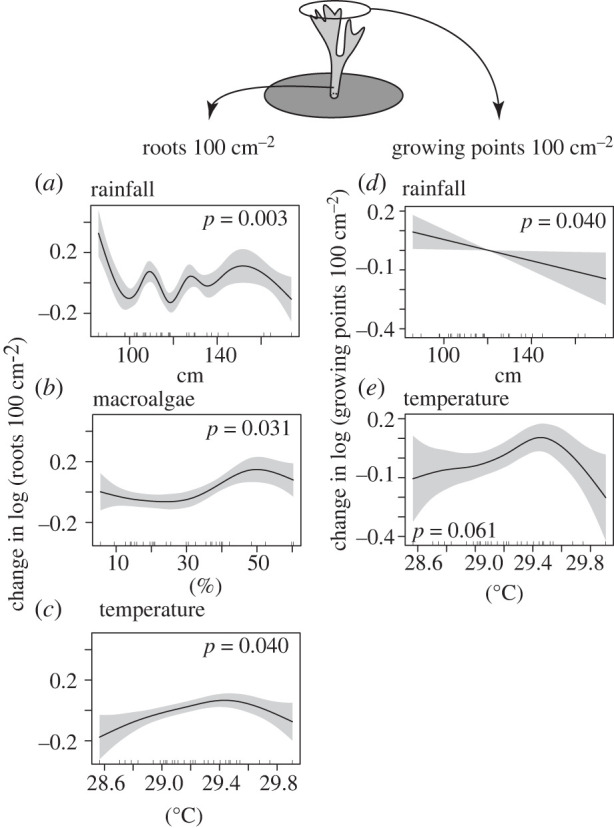
Results of GAMs testing for relationships between two morphological features (log-transformed) of *Millepora* sp. and up to four predictors: hurricanes (qualitative effect), rainfall, macroalgal cover and temperature^1^ (mean over August–October) (quantitative effects). Plots display significant smoothed relationships (except for (*e*), *p* = 0.061) for the best-fit model for roots 100 cm^−2^ (*a*–*c*) and growing points 100 cm^−2^ (*d*,*e*) (table 1; electronic supplementary material, table S1). Abscissas show units of the predictors with rugs (internal ticks) showing the data, and ordinates display the change in the logarithm of the morphological features. Shaded belts are 95% confidence intervals. (Online version in colour.)

In the analyses of roots and growing points using GAMs, the values for 1995 were excluded as statistical outliers, and all other values were log-transformed to restore normality. For roots 100 cm^−2^, the best-fit relationship was obtained with a model including hurricanes, rainfall, temperature^1^ and macroalgal cover (table 1; electronic supplementary material, table S2). This model explained 79% of the variation and included significant smoothed, curvilinear components for all three quantitative predictors; the effect of hurricanes was not significant. These relationships showed that high rainfall was associated with larger sheets relative to roots (i.e. the quotient declined), macroalgal cover ≥ ∼40% was associated with more roots relative to the size of sheets, and high temperature was associated with more branches relative to the size of sheets (table 1 and figure 3*a–c*). For growing points 100 cm^−2^, the best-fit relationship was obtained with three quantitative predictors that explained 30% of the variation (table 1; electronic supplementary material, table S2). Rainfall had a significant linear effect in explaining growing points 100 cm^−2^, with high rainfall associated with larger sheets relative to growing points, and there was a trend for high temperature to be associated with more growing points relative to the size of sheets. The relationship with macroalgal cover was not significant (table 1 and figure 3*d,e*).

**Table 1.** Results of GAMs testing for the effects of three quantitative variables (rainfall, macroalgal cover and temperature^1^), and one qualitative variable (hurricanes) on roots and growing points (figure 1) of *Millepora* sp. The best model was selected based on ΔAIC_c_ and *R*^2^. Asterisk = results of significant smoothed terms (and trends, ^†^) displayed in figure 3.

dependent variable	type				
roots 100 cm^2^	*parametric coefficients*	estimate	s.e.	*t*	*p*
	intercept	1.039	0.013	78.333	<0.001
	hurricanes	−0.031	0.046	0.671	0.514
	*smoothed terms*	edf	ref d.f.	*F*	*p*
	rainfall (cm)	7.390	8.031	5.507	0.003*
	macroalgae cover (%)	3.817	4.577	3.419	0.031*
	temperature^1^ (°C)	2.936	3.583	3.425	0.040*
	ΔAIC_c_	0.000			
	*R*^2^	0.786			
growing points 100 cm^2^	*parametric coefficients*	estimate	s.e.	*t*	*p*
	intercept	1.862	0.024	78.928	<0.001
	*smoothed terms*	edf	ref d.f.	*F*	*p*
	rainfall (cm)	1.000	1.000	4.716	0.040*
	macroalgae cover (%)	1.000	1.000	1.817	0.191
	temperature^1^ (°C)	3.352	4.148	2.601	0.061^†^
	ΔAIC_c_	0.000			
	*R*^2^	0.299			

## 4. Discussion

Understanding how organisms respond to environmental conditions is a central objective of ecology [40]. For sessile taxa on hard surfaces, varying conditions bring challenges that differ from those experienced by vagile organisms, because they are unable to move once they have settled. The morphological strategies adopted by organisms and, variation therein, provide an important means to address the limitations of immobility, and taxa that can exploit multiple strategies can have selective advantages [1]. This study used a calcareous hydrocoral to test for beneficial consequences of a sheet-tree morphology [1] for a sessile taxon on a crowded and disturbed coral reef. Three decades of abundance records revealed apparent ecological anarchy [41] in the dynamics of *Millepora* sp*.*, yet this variation was mediated by a plastic sheet-tree morphology that enhanced ecological success. In response to spatial competition and high temperature, *Millepora* sp. increased its relative reliance on trees, and in response to hydrodynamic forces (i.e. storms), it increased reliance on sheets. These results underscore the selective value of morphological design in an important functional group of benthic space holders confronted with the challenges of present-day environmental conditions.

*Millepora* spp. are ecologically important on coral reefs [13,14] and have been studied for greater than 135 years [42]. Yet even with more contemporary interest in their biology, they remain the topics of only approximately 19 publications yr^−1^ [14]. Scleractinians are the subjects of numerous time series analyses, but *Millepora* spp. are rarely a focus of monitoring [14]. The eastern Pacific provides one exception to this trend where decades of research allowed the detection of a near-population extirpation in 1990 through bleaching [43,44]. The San Blas Islands of Panama provide another example, and here *Millepora* cover was relatively stable from 1983 to 1990 [45]. In St John, annual photoquadrats from 1992 to 2008 at six sites (one of which sites supports the present study) characterized *Millepora* sp. as a rare taxon whose cover remained less than 1.5% but varied among years [46]. Much of the variation in *Millepora* sp. abundance could not be explained by environmental conditions, although the analysis showed that sheets increased in size following cool winters, and branches were removed by storms [46]. The present study focused on Cabritte Horn where *Millepora* sp. has been abundant for decades, occupying in 1985 up to approximately 55% of the benthos at 4 m depth and approximately 12% at 12 m depth, in both cases approximately 30 m from the transect described herein [30]. Thirty years of data again revealed variation in *Millepora* sp. abundance [46] (figure 2; electronic supplementary material, figure S1), but for the first time, they shows how a sheet-tree morphology can be used to benefit from prevailing environmental conditions, and the consequences thereof. Some of the variation in *Millepora* sp. abundance is consistent with expectations, for instance, the declines in size of sheets in 1999 and 2005 following high temperatures to which *Millepora* sp. is susceptible [14,43] and the loss of branches over 1995–1996 and 2017–2018 due to storms [18]. The increases in size of sheets following cool years are similar to previous results [46], and following hurricanes they probably reflect the pre-emption of vacant space created by physical disturbance [30]. Overall, however, the graphical display of three decades of *Millepora* sp. abundance still appears characterized by erratic changes.

A theme in decades of *Millepora* research has been the remarkable corallum morphology, which typically consists of encrusting sheets and trees that are plastic in response to environmental conditions [13,14,17]. Delicately branched colonies occur in calm water, sturdily branched colonies in turbulent conditions and encrusting sheets are found at sites with the roughest conditions and turbid water [17]. These trends help to explain why *Millepora* spp. can spatially dominate in shallow and rough conditions [30]. The wide range of morphologies adopted by *Millepora* can be expressed even within a clonal genotype [47], and the high growth rates of *Millepora* indicate the ease with which changes in morphology can be accomplished. Sheets, for example, can spread at up to 1977 cm^2^ y^−1^ [48], with a mean rate of 28 cm^2^ y^−1^ on vertical surfaces [18], and from these sheets, branches grow at 2.9–14.0 mm y^−1^ [18,48,49], and new branches are produced at approximately 0.7 branches 100 cm^2^ y^−1^ [18]. Where the functional underpinnings of *Millepora* success have been addressed, their sheet-tree morphology often is cited as a contributing factor [13,14,17,18]. However, despite frequent reference to the selective value of a sheet-tree morphology for *Millepora*, the benefits of exploiting this strategy have not been demonstrated, and it rarely has been placed in the broader context advocated by JBC Jackson [1].

This study describes how the morphology of *Millepora* sp. changed in association with environmental conditions. Given that low values of the quotients used to characterize the sheet-tree morphology indicate greater relative exploitation of sheets versus trees, the GAMs suggest that sheets are favoured by low temperature, low cover of macroalgae and high rainfall; trees (roots and growing points) are favoured by low rainfall, high macroalgal cover and a mean temperature of approximately 29.5°C. While it is premature to ascribe causation to these associations, the biology of *Millepora* sp. provides clues (figure 1*b*) to the underlying processes that might be important.

Sheets support the acquisition of benthic space when food is abundant as a result of zooplanktivory and photoautotrophy at high light intensities [13,14]. Large sheets exclude recruitment by other taxa and reduce the susceptibility to biotic interactions (e.g. competition) through a low ratio of colony circumference to surface area [1]. They are also resistant to accelerational seawater flow [50] and consequently persist through storms [14,18], after which they can pre-empt vacant space through rapid growth [49]. Sheets can, however, be forced to retreat by spatial competitors such algae [49,51], including the peyssonnelid algal crust that became abundant at Cabritte Horn after approximately 2012 [52]. They can also be reduced in size by bleaching under elevated temperature and high light [14,43], which indicates that these effects would be more common in shallow water and on upward-facing surfaces. Expression of the sheet-tree morphology of *Millepora* sp. therefore should vary across gradients of environmental conditions, for example, from shallow to deep water through depth-dependent reductions in wave energy and the quantity of light, and over time as conditions change.

Together, the present results suggest that sheeting in *Millepora* sp. is associated with: (i) elevated rainfall, which could reflect effects of multiple indirect pathways including reduced thermal bleaching under attenuated light [53], the benefits of bigger sheets in harvesting light for photosynthesis and autotrophy [14], and enhanced heterotrophic feeding on plankton and particulates that can be associated with terrestrial run-off [35]; (ii) low macroalgal cover that could facilitate space pre-emption by *Millepora* sp. sheets [30] and (iii) lower temperatures, which alleviates thermal bleaching, particularly for upward-facing sheets exposed to light. By contrast, tree morphologies represent commitment to the settlement location, access to resources in the water column, and a means to escape conditions on the substratum [1]. Further, detached branches serve as asexual propagules [54], and by elevating reproductive structures above the benthos, can enhance dispersal [55]. Thus, trees in *Millepora* sp. are likely to be favoured by stringent benthic competition (e.g. with macroalgae), elevated temperatures favouring bleaching in sheets, and low rainfall associated with reduced cloud cover and high light intensities that promote calcification in support of the production of branches [36,56].

Since storms waves break *Millepora* sp. branches [13,14,18], and then favour the expansions of sheets (e.g. in 2018, figure 2), it is surprising that hurricanes were not significant in the GAM analyses. This may have been related to their small effect size on *Millepora* sp. abundance, the absence of continuous measures of the underwater forces of storm waves and the lack of tagged colonies [18]. Without tagged colonies, the causes of increases in the quotients characterizing the sheet-tree morphology could not be determined, notably the extent to which they were caused by the growth of new branches versus shrinkage of sheets. Nevertheless, the results make the case that multiple transitions between relative reliance on sheets versus trees over an ecologically relevant time scale facilitate the success of a sessile animal characterized by a plastic, sheet-tree morphology.

This study highlights how an ecologically important group of organisms, unified by immobility as adults, exploits their morphological strategy to alleviate the limitations of being sessile under contemporary environmental conditions. For the subset of these organisms that inhabit hard substrata in the marine environment, Jackson [1] codified their morphological strategies in a framework that has influenced the analysis of form and function for decades [50,57]. Here, one of these morphological strategies is used as a lens through which changes in abundance of a common reef coral are interpreted, thus revealing the waxing and waning of reliance on sheets versus trees that has contributed to their ecological success.

## Data Availability

Electronic supplementary material is available online [58]. All data supporting the present analyses are available online at bco-dmo: percent cover of *Millepora* at Cabritte Horn (doi:10.26008/1912/bco-dmo.875524.1), Abundance and percent cover of macroalgae at Cabritte Horn (doi:10.26008/1912/bco-dmo.875543.1), Daily seawater temperature at Yawzi Point (doi:10.26008/1912/bco-dmo.875694.1), Morphology and features of *Millepora* colonies at Cabritte Horn (doi:10.26008/1912/bco-dmo.875553.1).
